# 

**Published:** 1892-01-30

**Authors:** 


					SJThomas s Hospital
'MKTOlR^oNOR iy/ch hospital
Ql a scotr Western Infirmary
WITH EXTRA NURiING .SUPPLEMENT.
Saturday, January 30th, 1892
[One Penny,

				

